# Beneficial effects of exercise on offspring obesity and insulin resistance are reduced by maternal high-fat diet

**DOI:** 10.1371/journal.pone.0173076

**Published:** 2017-02-24

**Authors:** Juliane Kasch, Sara Schumann, Saskia Schreiber, Susanne Klaus, Isabel Kanzleiter

**Affiliations:** Department Physiology of Energy Metabolism, German Institute of Human Nutrition Potsdam-Rehbruecke, Nuthetal, Germany; Universidade do Estado do Rio de Janeiro, BRAZIL

## Abstract

**Scope:**

We investigated the long-term effects of maternal high-fat consumption and post-weaning exercise on offspring obesity susceptibility and insulin resistance.

**Methods:**

C57BL/6J dams were fed either a high-fat (HFD, 40% kcal fat) or low-fat (LFD, 10% kcal fat) semi-synthetic diet during pregnancy and lactation. After weaning, male offspring of both maternal diet groups (mLFD; mHFD) received a LFD. At week 7, half of the mice got access to a running wheel (+RW) as voluntary exercise training. To induce obesity, all offspring groups (mLFD +/-RW and mHFD +/-RW) received HFD from week 15 until week 25.

**Results:**

Compared to mLFD, mHFD offspring were more prone to HFD-induced body fat gain and exhibited an increased liver mass which was not due to increased hepatic triglyceride levels. RW improved the endurance capacity in mLFD, but not in mHFD offspring. Additionally, mHFD offspring +RW exhibited higher plasma insulin levels during glucose tolerance test and an elevated basal pancreatic insulin production compared to mLFD offspring.

**Conclusion:**

Taken together, maternal HFD reduced offspring responsiveness to the beneficial effects of voluntary exercise training regarding the improvement of endurance capacity, reduction of fat mass gain, and amelioration of HFD-induced insulin resistance.

## Introduction

In the last decades, obesity became a serious health problem in developed countries and it is increasing worldwide with further industrialization. Consequently, the number of obese women in reproductive age is rising [1]. This is of particular importance, since maternal pre-pregnancy BMI is a predictor of childhood obesity [2, 3] which in turn is a major risk factor for adult obesity and early type 2 diabetes development [4].

Obesity is thought to be the consequence of unhealthy nutrition and a lack of physical activity. Hence, reducing maternal obesity by nutritional changes or exercise may help to attenuate the burden of the disease in future generations. To highlight the importance of perinatal maternal nutrition for offspring health, it was demonstrated in rodents that low-fat diet (LFD) feeding of pregnant obese dams ameliorates offspring metabolism [5, 6]. Furthermore, regular exercise during pregnancy improved body fat mass development, glucose tolerance and increased glucose clearance in various tissues/organs of adult offspring [7–9].

Several studies have focused on the impact of maternal high-fat diet (mHFD) consumption on the development of non-alcoholic fatty liver disease (NAFLD) in the offspring [10–13]. Lately, the skeletal muscle as a key insulin-responsive tissue is receiving more and more attention [9, 14–16]. This is of particular importance, since skeletal muscle represents about 40% of the whole body mass, highlighting it as an essential tissue for glucose and fatty acid utilization. Furthermore, the fetal state is of crucial importance for skeletal muscle development because there is no increase in muscle fiber numbers after birth [17].

Bayol and colleges already demonstrated that unhealthy perinatal maternal nutrition reduces the offspring’s muscle force [18]. In addition, we showed recently that mHFD consumption during pregnancy and lactation impaired exercise performance in young adult offspring [15]. We now hypothesize that this compromised muscle function in offspring has long-term effects on the development of diet induced obesity and might also impair the beneficial effects of exercise on body composition and glucose homeostasis.

Hence, in the present study we investigated the long-term interaction between mHFD feeding and post-weaning exercise, focusing on its potential to affect obesity development and glucose homeostasis in the offspring. More precisely, we aim to scrutinize the effects of perinatal mHFD consumption independent of maternal obesity. High-fat diet (HFD) feeding of the dams started three days prior to mating and continued during gestation and lactation; a maternal LFD (mLFD) group received a LFD during this time. After weaning, male offspring of both groups received a LFD to ensure uniform growth conditions during adolescence. From week (wk) 7 onwards, half of each group was given access to a running wheel (RW) to investigate the effects of post-weaning exercise. To assess the effects of perinatal nutrition and post-weaning exercise on offspring obesity predisposition in adulthood, HFD feeding was performed from wk 15 until wk 25. Endurance capacity was determined at wk 7, 15, and 25 to evaluate training efficiency. We hypothesized that maternal HFD feeding during gestation and lactation decreases offspring endurance capacity as well as training efficiency, and thus increases obesity susceptibility in adulthood.

## Methods

### Animals and experimental set up

Animal experiments were approved by the ethics committee of the Ministry for environment, health, and consumer protection (State Brandenburg, Germany, Permission no.GZ-23-2347-26-2010).

Mice were kept in a light- and temperature-controlled facility at 22°C with a 12h light-12h dark cycle. Starting three days prior mating, dams (C57BL/6, 8 wks old) received either a HFD (40% energy from fat, 23% energy from protein and 37% energy from carbohydrate; Research Diets Services) or LFD (10% energy from fat, 23% energy from protein, 67% energy from carbohydrate; Research Diets Services) during pregnancy and lactation [15]. Since standard chow diets usually contain poorly specified ingredients that can strongly vary in their composition between batches and between providers, all diets used in the present study were semi-synthetic with a defined nutrient composition as recommended for nutritional physiology studies [19]. Overall, the fat content of the LFD was comparable to a standard maintenance chow which has 12% energy from fat (Type 1320 diet; Altromin, Germany). Importantly, the semi-synthetic diets used in this study had a defined fat composition (70% sunflower, 18% coconut and 12% faxseed oil) while the fat of a standard chow is usually not further specified.

**Fig 1:**
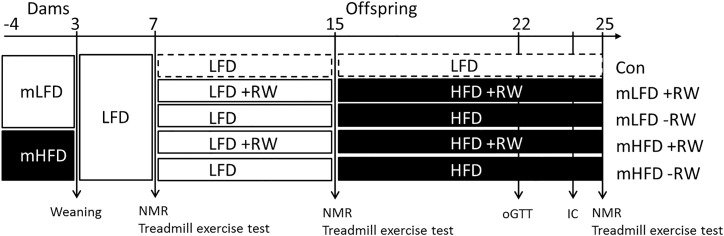
Experimental study design. Dams were either fed a low-fat (LFD) or a high-fat diet (HFD) before and throughout pregnancy and lactation. After weaning, male offspring received LFD until an age of 15 wks. At an age of 15 wks, mice received HFD for 10 wks. Half of each group got access to a running wheel (RW) as voluntary exercise training. As a control (baseline) group (Con), mLFD offspring without RW access were fed LFD during the whole experiment. Body composition and treadmill exercise capacity were measured in wk 7, 15, and 25. A four-hour oral glucose tolerance test (oGTT) was performed in wk 22 and indirect calorimetry (IC) was performed in wk 24.

Two females were introduced to one male for one wk and subsequently individually caged with a total of 24 and 18 dams in the mLFD and mHFD group, respectively. Initial litter size was not affected by maternal diet. The individual litter size was adjusted to 5–7 pups, with no more than 1–2 male siblings finally present in one experimental group. Post-weaning male offspring were single caged and received LFD until an age of 15 wks. At an age of 7 wks, half of each group got access to a RW (TSE Systems, Bad Homburg, Germany) as voluntary exercise training and at an age of 15 wks, all mice received HFD until the end of the study (25 wks). This resulted in four respective groups: mLFD-RW, mLFD+RW, mHFD-RW, mHFD+RW (Fig 1). As an additional control group (Con), mLFD offspring without RW and HFD intervention (mLFD-RW LFD) were used to obtain baseline values. All mice were sacrificed in wk 25 after 2h of fasting and 24h after the last treadmill exercise bout. The animals were euthanized with isoflurane and peripheral blood was taken by cardiac puncture. Tissues were removed, weighed and immediately frozen in liquid nitrogen before they were stored at -80°C.

### Body composition

Body composition was determined in wk 7, 15 and 25 by quantitative nuclear magnetic resonance spectroscopy (NMR; Minispec MQ10 NMR Analysis Bruker, Karlsruhe, Germany) as described previously [20]. Lean mass was calculated by subtracting body fat mass (obtained by NMR) from body mass.

### Indirect calorimetry

In wk 24, energy expenditure and respiratory quotient (RQ) were measured by indirect calorimetry (IC) over a period of 23h using PhenoMaster System (TSE Systems GmbH, Homburg, Germany) as described [20].

### Endurance exercise capacity and training efficiency

Daily wheel-running and spontaneous cage activity were determined by IR motion detectors (TSE Systems GmbH, Homburg, Germany) and indicated no differences between mLFD and mHFD offspring. Endurance exercise capacity was determined on a six-lane treadmill (Columbus Instruments, Columbus, USA) at an age of 7 wks (before RW access), 15 wks (before HFD feeding), and 25 wks (at the end of the experiment). The starting speed of 5 m/min was increased every 5 min until a maximum speed of 28 m/min was reached. Mice ran until exhaustion or a maximum of 100 min. Exhaustion was defined as the inability of mice to resume running as evident by continuous contact (>5 sec) with the electrical shock grid at the end of each running lane. Running time was measured and the distance was calculated as the product of time and speed of the treadmill. Training efficiency was calculated as the difference in distance between the first, second and third treadmill test.

### Oral glucose tolerance test

After 16h of fasting, a four-hour oGTT was performed in wk 22. Following oral glucose application by gavage (2 mg glucose/g body weight), glucose concentrations were measured in tail vein whole blood by ContourXt glucose sensor (Bayer AG, Leverkusen, Germany) before glucose application and 15, 30, 60, 120, and 240 min after gavage. Plasma insulin was measured at baseline, 15 min, and 30 min after glucose administration and determined by Insulin Mouse Ultrasensitive ELISA (Alpco Diagnostics, Salem, USA).

### Pancreatic insulin measurement

For pancreatic insulin analytics, whole pancreas was homogenized in 1 mL ice-cold acid ethanol (0.18 M HCl in 70% ethanol) for 5 min at maximum speed. Insulin was extracted over night at 4°C. Samples were centrifuged (5000xg for 15 min at 4°C) and insulin concentrations were determined in the supernatants by Insulin Mouse Ultrasensitive ELISA.

### Quantitative real-time PCR

To analyze gene expression, RNA isolation and quantitative real-time PCR were performed as described previously [21]. Expression was calculated as ddCT using *ribosomal protein L13A* (*Rpl13a*) in skeletal muscle and *beta-2-microglobulin* (*B2m*) in liver for normalization; the Con group (mLFD -RW LFD) was set to a value of 1. Primer specificity was confirmed by melting curve analysis and agarose gel electrophoresis of the PCR reactions. The oligonucleotide sequences of all primers are listed in S1 Table.

### Western blot analysis

Protein was extracted from M. quadriceps (50 mg). SDS-PAGE, immunoblotting and chemoluminescence detection were performed as described before [22]. For Western Blotting, oxidative phosphorylation (OXPHOS) antibody (MitoSciences, MS604-300) was used. Protein expression was normalized to voltage-dependent anion-selective channel protein 1 (VDAC1; Cell Signaling, Danvers, USA).

### Triglyceride and glycogen analysis

Triglyceride concentrations were analyzed in M. quadriceps and liver as previously described [21]. Briefly, 40 mg tissue were homogenized in either 400 μL (muscle) or 800 μL (liver) of 10 mM sodium hydrogen phosphate buffer (pH 7.4; 1 mM EDTA, 1% polyoxyetylene-10-tridecyl ether). Samples were centrifuged at 23100xg for 30 min. The supernatant was incubated for 5 min at 70°C, stored on ice for 5 min and centrifuged again. Triglyceride content (Triglyceride Determination Kit, Sigma Aldrich, Steinheim, Germany) and protein concentrations (DC Protein Assay; Bio-Rad Laboratories GmbH, München, Germany) were measured in triplicates in the supernatants according to manufacturer’s instructions. Measurement of muscle and liver glycogen content was performed as described previously [15]. Briefly, 35 mg liver and 50 mg muscle were homogenized in 750 μL and 1000 μL of 0.1 M NaOH, respectively. The homogenate was incubated for 45 min at 70°C, stored on ice for 5 min and centrifuged (12400xg, 10 min, 4°C). Glycogen contents were determined in triplicates using the Starch Kit (R-Biopharm AG, Darmstadt, Germany). Concentrations were normalized to the protein content (DC Protein Assay; Bio-Rad Laboratories GmbH, München, Germany).

### Plasma analysis

Plasma free fatty acids, triglycerides, and cholesterol were measured according to published protocols [23].

### Citrate synthase activity

Citrate synthase activity was measured in M. quadriceps as previously described [24]. Briefly, muscle tissue was homogenized in 50 mM Tris, 1 mM EDTA (pH 7.4), and 0.1% Triton X-100 and subsequently centrifuged (13000xg, 10 min, 4°C). Ten μl of the supernatant were mixed with 215 μl of reaction buffer (100 mM Tris, 1 mM MgCl2, 1 mM EDTA (pH 8.2), and 0.1 M DTNB) and 25 μl of acetyl-CoA (3.6 mM). To start the reaction, 50 μl of oxaloacetate (3 mM) were added and the absorbance change at 412 nm was measured for 10 min at 37°C. Citrate synthase activity was calculated from the slope of the linear portion of the absorbance curve and normalized to the protein content (DC Protein Assay; Bio-Rad Laboratories GmbH, München, Germany). All analyses were completed in triplicates in a 96 well plate.

### Statistical analysis

Statistics were performed using SPSS (version 20; IBM SPSS Inc.) and GraphPad Prism 6 (GraphPad Software, Inc.) between all HFD-fed offspring groups; the Con group was always excluded from statistical analysis. As indicated in the figure legends, data were analyzed by unpaired t-test or two-way ANOVA with Bonferroni post hoc test. If the dataset failed normality test (Kolmogorov-Smirnov-test), Kruskal Wallis and Dunn´s multiple comparison test was conducted instead of two-way ANOVA. For correlation analysis, Spearman correlation was used. Statistical significance between the groups is indicated as: *p<0.05, **p<0.01 and ***p<0.001; in Figures with both, # and *, # indicates significant differences between the maternal diets (mLFD and mHFD), whereas * indicates significant RW effects (-RW vs. +RW within one maternal diet group). Normally distributed data are presented as mean with standard error (SE); non-normally distributed data as Tukey’s Boxplot using the median and the 25th to 75th percentile interval.

## Results and discussion

### Maternal high-fat diet promotes offspring obesity and impairs the effect of exercise on fat mass gain

To elucidate the effects of perinatal maternal HFD consumption (without prior maternal obesity) dams were fed a HFD (40% kcal fat) or LFD (10% kcal fat) throughout pregnancy and lactation, starting three days prior to mating. As indicated in S1 Fig, dams fed a HFD had a slightly (non significantly) increased body weight at weaning but did not develop obesity, probably due to the restricted time (less than 4 weeks) of HFD exposure. It is interesting to elucidate if the perinatal consumption of an unhealthy HFD alone leads to detrimental effects on the offspring. This would highlight the importance of a healthy nutrition during pregnancy and lactation, independent of the maternal phenotype i.e. obesity prior to conception.

**Fig 2:**
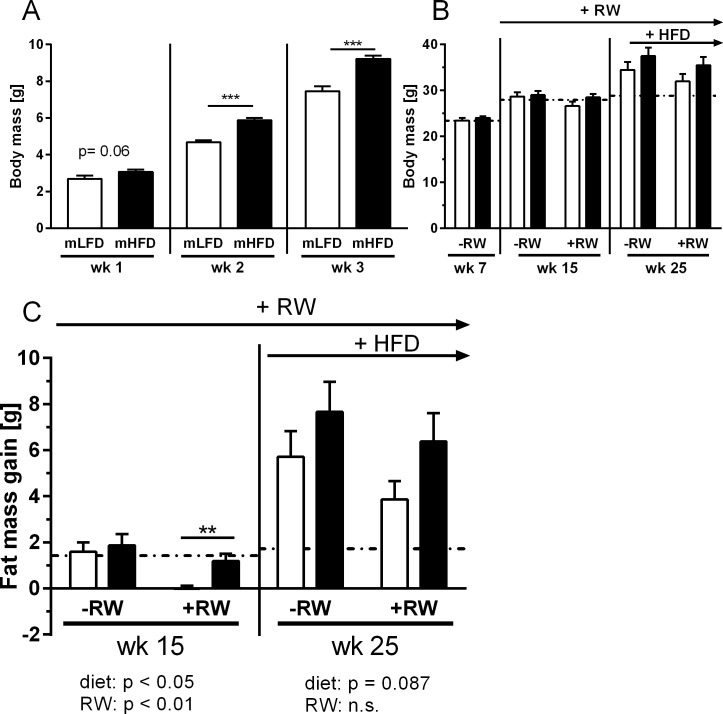
**Impact of maternal high-fat consumption on offspring body weight (A-B) and body fat gain (C).** Dams were either fed a low-fat (LFD) or a high-fat diet (HFD) before and throughout pregnancy and lactation. Maternal low-fat diet (mLFD; white bars) or maternal high-fat diet (mHFD; black bars) offspring were fed a LFD after weaning throughout an age of 15 wks. Afterwards they received HFD for 10 wks. Half of each group had access to a running wheel (RW) for voluntary training. As a control (baseline) group (Con), mLFD offspring without RW were fed a LFD during the whole experiment (dotted line). (C) Shown is the gain of body fat from wk 7 to wk 15 and wk 7 to wk 25. Data are mean + SE; (A) n = 17-36; (B/C) wk 7: n = 21–28; wk 15/25: n = 10-15. Data were analyzed using two-way ANOVA (Bonferroni post hoc test) and unpaired t-test with Welch’s correction (A/C).

While mHFD feeding did not significantly affect body weight of dams at weaning, offspring body weight was increased in the mHFD group between wk 1 and wk 4 (Fig 2A). Despite these initial differences, later body weight was similar in mLFD and mHFD offspring (Fig 2B). The fat mass increase from wk 7 to wk 15 was also similar in mLFD and mHFD offspring without RW. Interestingly, RW training completely prevented this fat gain in mLFD, but not in mHFD offspring (wk 15; Fig 2C). Remarkably, daily wheel-running and spontaneous activity were not different between the groups, suggesting that mHFD did not lead to a more “lazy” offspring phenotype. The preventive effect of RW training on fat mass gain was not observed upon 10 wks of HFD feeding (wk 25). Here, in both maternal groups, voluntary training could not reduce the HFD-induced fat mass gain. However, mHFD offspring showed a higher body fat mass than mLFD mice (Table 1) and also, in tendency, an increased fat mass gain after 10 wks of HFD feeding (Fig 2C). Lean mass, plasma parameters as well as energy expenditure and RQ were not affected by maternal diet or post-weaning exercise (Table 1). That voluntary training is not sufficient to completely reverse the negative effects of HFD feeding was also shown by Rajia et al. [16]. They described beneficial effects of exercise training on the offspring of chow- and HFD-fed dams and reported that exercise almost completely reversed the negative metabolic effects of maternal obesity in chow-fed, but not in HFD-fed offspring. Furthermore, even a more efficient treadmill exercise training provided only modest protection against HFD-induced body weight and fat mass gain [25].

**Table 1:** Offspring biometric data, energy metabolism and plasma parameters.

		No exercise (-RW)		Exercise (+RW)		p-Value	
	Con	mLFD	mHFD	mLFD	mHFD	mDiet	RW
**Biometric data**							
Lean mass at wk 7 [g]	20.35 ± 0.75	21.74 ± 0.81	21.57 ± 0.41	20.76 ± 0.79	22.05 ± 0.52	n.s.	n.s.
Fat mass at wk 7 [g]	1.20 ± 0.10	1.10 ± 0.08	1.35 ± 0.09	1.41 ± 0.11	1.27 ± 0.08	n.s.	n.s.
Lean mass at wk 15 [g]	23.30 ± 0.59	24.66 ± 0.62	24.40 ± 0.45	23.87 ± 0.75	24.57 ± 0.46	n.s.	n.s.
Fat mass at wk 15 [g]	2.62 ± 0.35	2.68 ± 0.44	3.23 ± 0.49	1.42 ± 0.17	2.46 ± 0.33	0.057	<0.05
Lean mass at wk 25 [g]	24.37 ± 0.54	26.30 ± 0.75	26.44 ± 0.65	25.40 ± 0.83	26.43 ± 0.64	n.s.	n.s.
Fat mass at wk 25 [g]	2.98 ± 0.45	6.80 ± 1.14	9.67 ± 1.22	5.26 ± 0.82	7.65 ± 1.24	<0.05	n.s.
**Energy metabolism**							
EE [kJ/d]	55.5 ± 0.9	56.8 ± 1.7	57.8 ± 2.0	55.6 ± 2.3	56.4 ± 1.6	n.s.	n.s.
RQ	0.95 ± 0.01	0.84 ± 0.01	0.83 ± 0.01	0.86 ± 0.01	0.86 ± 0.01	n.s.	n.s.
**Plasma parameters**							
TG [mmol/L]	0.421 ± 0.054	0.402 ± 0.094	0.286 ± 0.063	0.460 ± 0.086	0.411 ± 0.082	n.s.	n.s.
NEFA [mmol/L]	0.662 ± 0.035	0.568 ± 0.084	0.669 ± 0.072	0.616 ± 0.065	0.562 ± 0.053	n.s.	n.s.
Chol [mg/dL]	200.4 ± 16.8	212.6 ± 21.5	227.1 ± 20.6	217.4 ± 31.5	200.2 ± 21.6	n.s.	n.s.

Maternal low-fat diet (mLFD) or maternal high-fat diet (mHFD) offspring were fed a LFD after weaning throughout an age of 15 wks. Afterwards they received HFD for 10 wks. Half of each group had access to a running wheel (RW) for voluntary training. As control group (Con) mLFD offspring -RW were fed a LFD. Data are mean ±SE, n = 6-12. Indirect calorimetry for determination of energy expenditure and RQ was performed in wk 24, plasma data are from wk 25 after sacrifice. Data were compared by two-way ANOVA (Bonferroni post hoc test).

**Fig 3:**
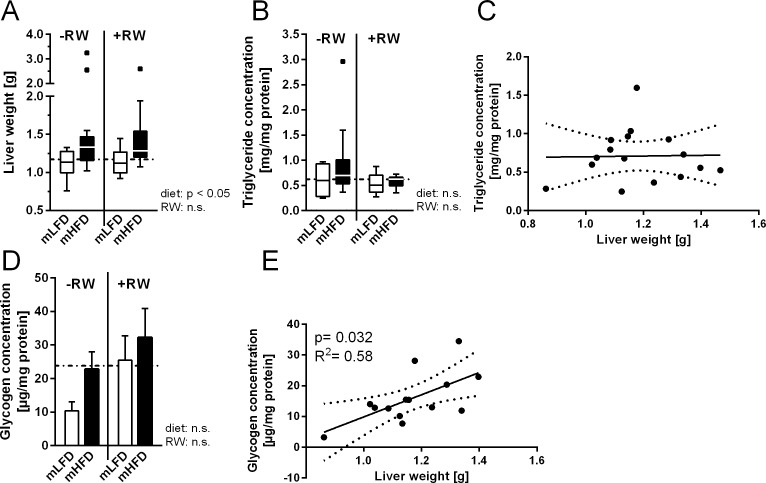
Impact of maternal high-fat intake on the offspring’s liver parameters. Maternal low-fat diet (mLFD) or maternal high-fat diet (mHFD) offspring were fed LFD after weaning throughout an age of 15 wks. Afterwards they received HFD for 10 wks. Half of each group had access to a running wheel (RW) for voluntary training. (A) offspring liver weight, (B/C) hepatic triglyceride concentration and its correlation with liver weight, (D/E) hepatic glycogen content and the respective correlation with liver weight. The dotted line in A/B/D indicates the level of the Con group (mLFD-RW LFD). Data are expressed as median with interquartile range (A/B) or mean +SE (D), (A) n = 10-15; (B) n = 6-12; (D) n = 4-11. Data were analyzed by Kruskal Wallis (Dunn´s test) or two-way ANOVA (Bonferroni post hoc test). (C/E) Spearman correlation analyses were performed with -RW animals.

A growing body of literature indicates a connection between maternal high-fat diet consumption and the development of a fatty liver in the offspring [10, 12, 13]. Indeed, we found that maternal HFD feeding increased offspring liver weight by about 40% independently of the activity level (Fig 3A). Surprisingly this was not the effect of an elevated hepatic triglyceride accumulation. While hepatic triglyceride content tended to be higher in mHFD -RW mice, liver triglycerides were not increased in the mHFD +RW group (Fig 3B). Furthermore, liver weight in the -RW groups did not correlate with the triglyceride content (Fig 3C) and hepatic gene expression of lipogenesis genes (*acetyl-CoA carboxylase alpha* (*Acaca*), *elongation of very long chain fatty acids 6* (*Elovl6*), *stearoyl-CoA desaturase-1* (*Scd1*) and *sterol regulatory element-binding transcription factor 1* (*Srebf1*)) was not altered (Table 2). On the other hand, liver glycogen content was also in tendency increased in mHFD -RW mice and was significantly correlated with liver weight (Fig 3D and 3E). This is in accordance with our previous study, where we showed that maternal HFD increases liver weight in LFD-fed offspring due to higher glycogen concentrations [15]. Nevertheless, this effect is contradictory to literature data reporting that the increased liver weight is the result of excessive hepatic lipid accumulation. Scd1, in particular, has been found in other studies to be associated with fatty liver development [10, 26]. The absence of any effect in our study could be explained by the use of different dietary fat contents/compositions. While many HFDs are composed of unrealistic 60% kcal fat [26], the Western-style diet used by Pruis et al. had a fat content of 45%, which mainly consisted of lard [10]. In contrast, the semisynthetic HFD in our study contained only 40% kcal from fat, which was composed of 70% sunflower oil, 18% coconut oil and 12% flaxseed oil [15]. Hence, the Western-style diet used by Pruis et al. [10] was composed mainly of saturated fatty acids, while the fat in our diet contained more healthy unsaturated fatty acids. Recently, Tsuduki et al. analyzed the effect of high maternal cholesterol intake on the offspring and found that it promotes fatty liver development [27]. Hence, cholesterol might be causal in maternal HFD-triggered fatty liver development by stimulating hepatic triglyceride accumulation in the offspring. Since the HFD in the present study was devoid of cholesterol, this could also explain the lack of hepatic triglyceride accumulation in the mHFD offspring.

**Table 2:** Hepatic gene expression of enzymes involved in lipogenesis.

	No exercise (-RW)		Exercise (+RW)		p-Value	
Gene	mLFD	mHFD	mLFD	mHFD	mDiet	RW
*Acaca*	0.763 ± 0.127	0.765 ± 0.104	0.631 ± 0.042	0.842 ± 0.164	n.s.	n.s.
*Elovl6*	0.806 ± 0.134	0.833 ± 0.274	0.645 ± 0.070	0.615 ± 0.133	n.s.	n.s.
*Scd1*	0.501 ± 0.080	0.501 ± 0.062	0.582 ± 0.101	0.498 ± 0.084	n.s.	n.s.
*Srebf1*	1.263 ± 0.286	0.967 ± 0.139	1.071 ± 0.124	1.368 ± 0.193	n.s.	n.s.

Maternal low-fat diet (mLFD) or maternal high-fat diet (mHFD) offspring were fed LFD after weaning throughout an age of 15 wks. Afterwards they received HFD for 10 wks. Half of each group had access to a running wheel (RW) for voluntary training. The control group (mLFD-RW LFD) was set to a value of 1. Data are mean ±SE, n = 6. Data were compared by two-way ANOVA (Bonferroni post hoc test).

Taken together, maternal HFD consumption during pregnancy and lactation increased body weight of young mice (until wk 4) and impaired the beneficial effects of RW training on fat mass gain (at wk 15). Furthermore, mHFD consumption increased liver weight which was not affected by voluntary exercise training.

### Maternal high-fat consumption impairs the beneficial effects of exercise training on HFD-induced insulin resistance

**Fig 4:**
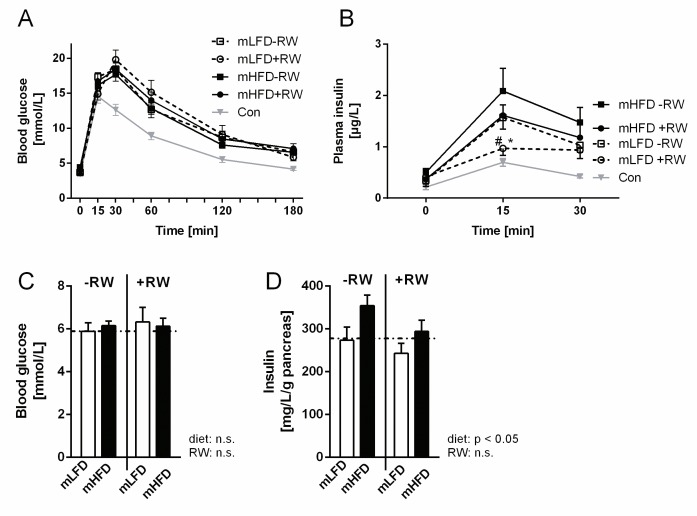
Impact of maternal high-fat diet intake on offspring HFD-induced insulin resistance. Maternal low-fat diet (mLFD) or maternal high-fat diet (mHFD) offspring were fed a LFD after weaning throughout an age of 15 wks. Afterwards they received a HFD for 10 wks. Half of each group had access to a running wheel (RW) for voluntary training. (A) glucose tolerance, (B) insulin sensitivity, (C) pancreas insulin content and (D) basal blood glucose concentration. The dotted line in C/D indicates the level of the Con group (mLFD-RW LFD). Data are mean ±SE (A) n = 8-15; (B) n = 10-15; (C) n = 5-6; (D) n = 6-12. Data were compared by two-way ANOVA (Bonferroni post hoc test). # indicates significant differences between the maternal diets (mLFD and mHFD), whereas * indicates significant RW effects (-RW vs. +RW).

While exercise improves insulin sensitivity in humans [28] and animals [29], maternal obesity is linked to hyperinsulinemia in the offspring [16, 30]. In order to investigate the interaction of both, an oGTT was performed in wk 22. While blood glucose levels did not differ between the groups (Fig 4A), plasma insulin levels 15 min after oral glucose application were reduced by RW in mLFD, but not in mHFD offspring (Fig 4B). This indicates that voluntary training has preventive effects on HFD-induced insulin resistance in mLFD offspring, only. As demonstrated in Fig 4C, mHFD offspring exhibited also increased pancreatic insulin levels, while blood glucose levels were not altered (Fig 4D). This again suggests the need for a higher beta-cell mediated insulin production to stabilize blood glucose levels in mHFD offspring. Gregorio et al. demonstrated that insulin production is increased in mHFD offspring and linked this effect to an altered pancreas morphology [31], which would also be interesting to analyze in our model.

Together, our data point out that mHFD offspring exhibit an increased pancreatic insulin production to counteract insulin resistance. Furthermore, voluntary exercise training has beneficial effects on HFD-induced insulin resistance in mLFD offspring only, indicating that maternal HFD consumption impairs this beneficial effect.

### Voluntary running wheel training improves endurance capacity of mLFD, but not mHFD offspring

**Fig 5:**
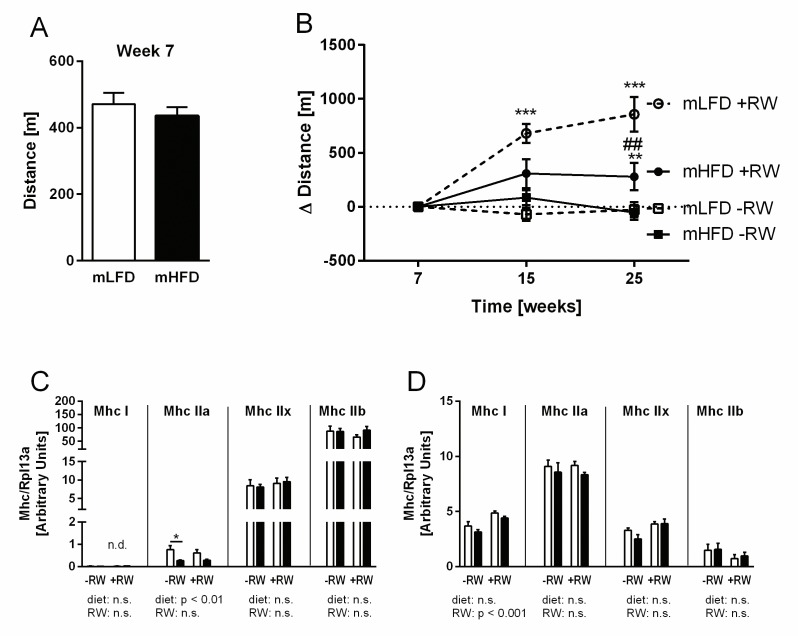
Impact of maternal high-fat feeding on offspring training efficiency and fiber type composition. Maternal low-fat diet (mLFD) or maternal high-fat diet (mHFD) offspring were fed a LFD after weaning throughout an age of 15 wks. Afterwards they received a HFD for 10 wks. Half of each group had access to a running wheel (RW) for voluntary training. (A) endurance capacity at wk 7, (B) training efficiency and (C/D) gene expression of myosin heave chain (Mhc) isoforms in M. quadriceps (C) and M. soleus (D). For Mhc gene expression, the Con group (mLFD-RW LFD) was set to 1. Data are mean ±SE; (A) n = 43-53; (B/C) n = 11-15; (C/D) n = 5-8. Data were compared by two-way ANOVA (Bonferroni post hoc test); # indicates significant differences between the maternal diets (mLFD and mHFD), whereas * indicates significant RW effects (-RW vs. +RW).

Along with our previous study in C3H mice [15], we demonstrate here in a different mouse strain that maternal HFD feeding decreases the effect of voluntary exercise training on offspring endurance capacity. While RW usage and endurance capacity at wk 7 (Fig 5A) were comparable between mLFD and mHFD offspring, RW exposure continuously increased exercise performance of mLFD offspring by about 160% from wk 7 to 15 and about 200% from wk 7 to 25 (Fig 5B). In mHFD offspring, RW exposure increased exercise performance non significantly by less than 70% from wk 7 to 15 with no further increase until wk 25 (Fig 5B). This clearly shows a long-term impairment of the offspring’s training efficiency by perinatal maternal HFD feeding.

We next investigated if offspring skeletal muscle characteristics were affected by maternal HFD. However, offspring muscle mass, glycogen and triglyceride content (S2 Fig) were not different between the groups. Endurance training triggers the skeletal muscle reorganization with a switch towards more oxidative fiber types that counteracts muscle fatigue by modifying substrate metabolism and contraction properties [15]. To investigate the effects of mHFD on skeletal muscle fiber type composition, we performed gene expression analysis of myosin heavy chain (Mhc) isoforms in M. quadriceps (fast twitch, glycolytic, type II) and M. soleus (slow twitch, oxidative,type I). Mhc gene expression profiling showed that M. quadriceps is composed almost exclusively of type II fibers with a clear abundance of *Mhc IIb* (Fig 5C) whereas M. soleus showed highest levels of *Mhc I* and *IIa* gene expression (Fig 5D). The effects of RW and maternal diet on Mhc composition were only marginal, with RW enhancing the expression of *Mhc I* (M. soleus) and mHFD reducing *Mhc IIa* (M. quadriceps). Nevertheless, mLFD offspring expressed (at least in tendency) higher levels of oxidative muscle fibers (*Mhc I; Mhc IIa*), which are known to be more pronounced in endurance athletes compared to sprinters [32].

We previously provided evidence that skeletal muscle lipogenesis and basal glucose uptake were significantly reduced by mHFD feeding in 12 wk old mice [15]. This suggests that the substrate availability during endurance exercise is impaired by mHFD consumption leading to a decreased exercise performance of mHFD offspring. To further elucidate this issue in the present study, gene expression in M. quadriceps was analyzed (S2 Table). We focused primarily on glucose metabolism, since glucose is an important energy substrate of the contracting muscle [33]. However, expression of the glucose transporters *Glut1* and *Glut4*, as well as important enzymes of glucose metabolism (*phosphofructokinase (Pfkm)*, *phosphoenolpyruvate carboxykinase 1 (Pck1))* were not differentially regulated between the groups. Only the expression of *pyruvate carboxylase (Pcx)* was reduced in mHFD offspring +RW, which could be a hint for a disturbed glucose metabolism.

**Fig 6:**
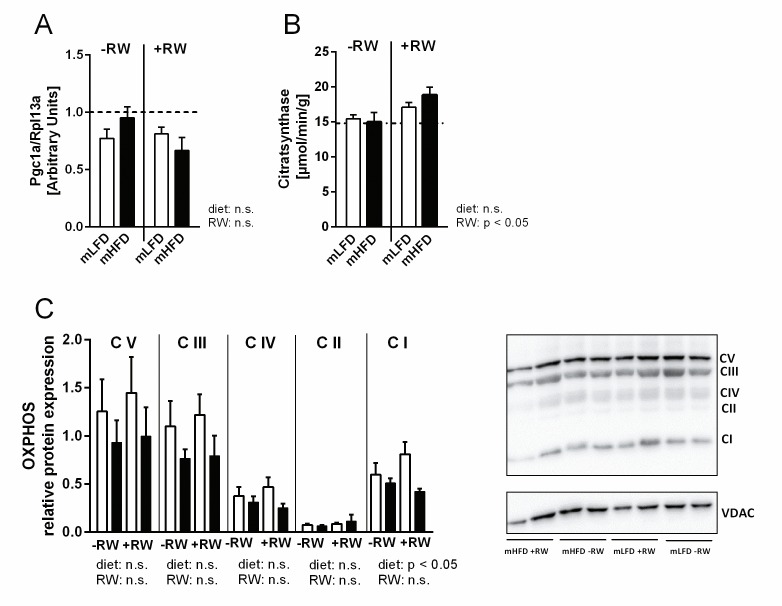
Effects of maternal high-fat consumption on offspring mitochondrial biogenesis in skeletal muscle. Maternal low-fat diet (mLFD) or maternal high-fat diet (mHFD) offspring were fed a LFD after weaning throughout an age of 15 wks. Afterwards they received a HFD for 10 wks. Half of each group had access to a running wheel (RW) for voluntary training. (A) Relative (ddCt) gene expression of *Pgc1α*, (B) citrate synthase activity and (C) protein expression of OXPHOS in M. quadriceps. The dotted line in A/B indicates the level of the Con group (mLFD-RW LFD). OXPHOS protein expression was normalized to VDAC (C). Data are mean +SE; (A) n = 6-8; (B) n = 6-7; (C) n = 5-6. Data were analyzed by two-way ANOVA (Bonferroni post hoc test).

Since gestational exercise prevents maternal HFD-induced peroxisome proliferator-activated receptor gamma coactivator 1-alpha (Pgc1α) hypermethylation and enhances its gene expression [34], *Pgc1α* is also an interesting target in the present study. However, we could neither detect a maternal nor a RW effect on *Pgc1α* expression (Fig 6A). Additionally, citrate synthase activity as a marker for mitochondria density was not affected by the maternal diet while it was significantly higher in the offspring with RW access (Fig 6B). The latter is in accordance with literature data reporting an induced oxidative mitochondrial capacity by physical exercise [35]. OXPHOS protein expression of complex I and in tendency also of complex III-V was decreased in mHFD offspring (Fig 6C). This suggests that maternal HFD might attenuate the ability of the electron transport chain to generate a proton gradient required for ATP production which is of particular importance for endurance exercise, and could at least partly explain the reduced training efficiency of mHFD offspring.

In summary, maternal HFD consumption negatively impacts the offspring’s response to voluntary exercise training by an impairment of endurance capacity. However, only slight molecular changes were detected in skeletal muscle which overall are unlikely to account for the pronounced differences in training efficiency and endurance capacity between mLFD and mHFD offspring. This might be due to the fact that the mice were sacrificed 24h after the last treadmill exercise bout. Exercise induced changes in glucose uptake and metabolism return to pre-exercise levels within 24h after exercise [33] and were therefore not detectible in the present study. It is possible that maternal HFD mainly affects the acute cellular responses to exercise. Hence, to study the underlying molecular mechanisms of the reduced exercise capacity of mHFD offspring, future studies should include a time course. By analyzing skeletal muscle in mice before, directly after and e.g. 3h after an exhaustive exercise bout, a clear conclusion about the metabolic changes in the skeletal muscle could be drawn.

In conclusion, we confirmed the concept that maternal HFD consumption (even without maternal obesity) increases the susceptibility of the offspring to diet-induced obesity and its associated disorders. Additionally, we showed that maternal HFD reduces the offspring’s responsiveness to the beneficial effects of exercise training regarding the improvement of endurance capacity, reduction of fat mass gain, and amelioration of HFD-induced insulin resistance. However, the exact molecular mechanisms for the observed effects remain to be elucidated. Preferably this could be investigated by performing a time course that detects the effects of an acute exercise bout. Additionally, epigenetic modifications should be analyzed in detail. Overall, this could be of importance for the development of appropriate anti-obesity strategies which may have long-term multigenerational effects.

## Supporting information

S1 Table Sequences of primers used for quantitative real-time PCR. (DOCX)

S2 Table Gene expression in M. quadriceps. Maternal low-fat diet (mLFD) or maternal high-fat diet (mHFD) offspring were fed a LFD after weaning throughout an age of 15 wks. Afterwards they received a HFD for 10 wks. Half of each group had access to a running wheel (RW) for voluntary training. The control group (mLFD-RW LFD) was set to a value of 1. Data are mean ±SE, n = 6–8. Data were compared by two-way ANOVA.
(DOCX)

S1 Fig Impact of maternal high-fat consumption on maternal body weight. Starting three days prior mating, dams were either fed a low-fat (LFD) or a high-fat diet (HFD) throughout pregnancy and lactation. Data are mean + SE; (A) n = 18-24. Data were analyzed using an unpaired t-test.
(TIF)

S2 Fig **Effects of maternal high-fat consumption on offspring muscle mass (A), triglyceride (B) and glycogen content (C).** Maternal low-fat diet (mLFD) or maternal high-fat diet (mHFD) offspring were fed a LFD after weaning throughout an age of 15 wks. Afterwards they received a HFD for 10 wks. Half of each group got access to a running wheel (RW) for voluntary training. The dotted line represents the Con group (mLFD-RW LFD). Data are mean with +SE; (A) n = 10–15; (B/C) n = 4–5. Data were analyzed by two-way ANOVA (Bonferroni post hoc test).
(TIF)
