# Correction: Beneficial effects of exercise on offspring obesity and insulin resistance are reduced by maternal high-fat diet

**DOI:** 10.1371/journal.pone.0175100

**Published:** 2017-03-28

**Authors:** Juliane Kasch, Sara Schumann, Saskia Schreiber, Susanne Klaus, Isabel Kanzleiter

The following information is missing from the Funding section: The publication of this article was funded by the Open Access Fund of the Leibniz Association.
