# Supplementary material for: Beneficial effects of exercise on offspring obesity and insulin resistance are reduced by maternal high-fat diet
Source: PLoS One. 2017 Feb 24;12(2):e0173076. doi: 10.1371/journal.pone.0173076 (PMC5325607; doi:10.1371/journal.pone.0173076)
Supplement: S1 Table — (DOCX) [file pone.0173076.s001.docx]

**S1 Table. Sequences of primers used for quantitative real-time PCR**

| **Gene** | **Gene ID** | **Forward primer** | **Reverse primer** |
| --- | --- | --- | --- |
| *Acetyl-CoA carboxylase alpha* | *Acaca* | TTTCACTGTGGCTTCTCCAG | TGCATTTCACTGCTGCAATA |
| *Beta-2-microglobulin* | *B2m* | CCCCACTGAGACTGATACATACGC | AGAAACTGGATTTGTAATTAAGCAGGTTC |
| *Elongation of very long chain fatty acids 6* | *Elovl6* | TGCAGGAAAACTGGAAGAAGTCT | AGCGGCTTCCGAAGTTCAA |
| *Solute carrier family 2 (facilitated glucose transporter), member 1* | *Glut1* | cgggtatcaatgctgtgttc | gtccagctcgctctacaaca |
| *Solute carrier family 2 (facilitated glucose transporter), member 4* | *Glut4* | CTATGCTGGCCAACAATGTC | CCCTGATGTTAGCCCTGAGT |
| *Myosin heavy chain I* | *Mhc I* | CCAAGAGCCGGGACATTG | TTGGAGCTGGGTAGCACAAGA |
| *Myosin heavy chain IIa* | *Mhc IIa* | GTCTGCGCAAACACGAGAGA | CCAAATCCTGAAGCCTGAGAATAT |
| *Myosin heavy chain IIx* | *Mhc IIx* | CAGATCGGGAGAACCAGTCT | CCTGCATTTTGCCAGAAGTT |
| *Myosin heavy chain IIb* | *Mhc IIb* | AACAGAAGCGCAACATCGAA | TTTAGTCTGTAGTTTGTCCACCAAGTC |
| *Phosphoenolpyruvate carboxykinase 1* | *Pck1* | TTTGTAGGAGCAGCCATGAG | TGATGATCTTGCCCTTGTGT |
| *Pyruvate carboxylase* | *Pcx* | tccgtgtccgaggtgtaaa | caggaactgctggttgttga |
| *Phosphofructokinase* | *Pfkm* | GCCGGCTCAGTGAGACAAG | GATGGCACCTTCAGCAACAAT |
| *Peroxisome proliferator-activated receptor gamma coactivator 1-alpha* | *Pgc1α* | CTACAGACACCGCACACACC | GCGCTCTTCAATTGCTTTCT |
| *60 S ribosomal protein L13a* | *Rpl13a* | GTTCGGCTGAAGCCTACCAG | TTCCGTAACCTCAAGATCTGCT |
| *Stearoyl-CoA desaturase-1* | *Scd1* | TTCTTCTCTCACGTGGGTTG | CGGGCTTGTAGTACCTCCTC |
| *Sterol regulatory element-binding transcription factor 1* | *Srebf1* | GAGGATAGCCAGGTCAAAGC | AGGATTGCAGGTCAGACACA |
