# Supplementary material for: Beneficial effects of exercise on offspring obesity and insulin resistance are reduced by maternal high-fat diet
Source: PLoS One. 2017 Feb 24;12(2):e0173076. doi: 10.1371/journal.pone.0173076 (PMC5325607; doi:10.1371/journal.pone.0173076)
Supplement: S2 Table — Maternal low-fat diet (mLFD) or maternal high-fat diet (mHFD) offspring were fed a LFD after weaning throughout an age of 15 wks. Afterwards they received a HFD for 10 wks. Half of each group had access to a running wheel (RW) for voluntary training. The control group (mLFD-RW LFD) was set to a value of 1. Data are mean ±SE, n = 6–8. Data were compared by two-way ANOVA. (DOCX) [file pone.0173076.s002.docx]

**S2 Table. Gene expression in M. quadriceps**

|  | **No exercise (-RW)** | | | **Exercise (+RW)** | | | **p-Value** | |
| --- | --- | --- | --- | --- | --- | --- | --- | --- |
| **Gene** | **mLFD** |  | **mHFD** | **mLFD** |  | **mHFD** | **mDiet** | **RW** |
| *Glut1* | 1.036 ± 0.07 |  | 0.986 ± 0.06 | 1.010 ± 0.03 |  | 0.977 ± 0.07 | n.s. | n.s. |
| *Glut4* | 0.837 ± 0.06 |  | 0.822 ± 0.02 | 0.879 ± 0.03 |  | 0.813 ± 0.04 | n.s. | n.s. |
| *Pck1* | 0.527 ± 0.11 |  | 0.368 ± 0.02 | 0.628 ± 0.13 |  | 0.588 ± 0.09 | n.s. | n.s. |
| *Pcx* | 0.706 ± 0.04 |  | 0.794 ± 0.08 | 1.107 ± 0.18 |  | 0.546 ± 0.04 | 0.03 | n.s. |
| *Pfkm* | 0.887 ± 0.06 |  | 1.039 ± 0.05 | 0.921 ± 0.06 |  | 0.778 ± 0.14 | n.s. | n.s. |

Maternal low-fat diet (mLFD) or maternal high-fat diet (mHFD) offspring were fed a LFD after weaning throughout an age of 15 wks. Afterwards they received a HFD for 10 wks. Half of each group had access to a running wheel (RW) for voluntary training. The control group (mLFD-RW LFD) was set to a value of 1. Data are mean +SE, n=6-8. Data were compared by two-way ANOVA.
