# Supplementary figures and images for: Beneficial effects of exercise on offspring obesity and insulin resistance are reduced by maternal high-fat diet
Source: PLoS One. 2017 Feb 24;12(2):e0173076. doi: 10.1371/journal.pone.0173076 (PMC5325607; doi:10.1371/journal.pone.0173076)

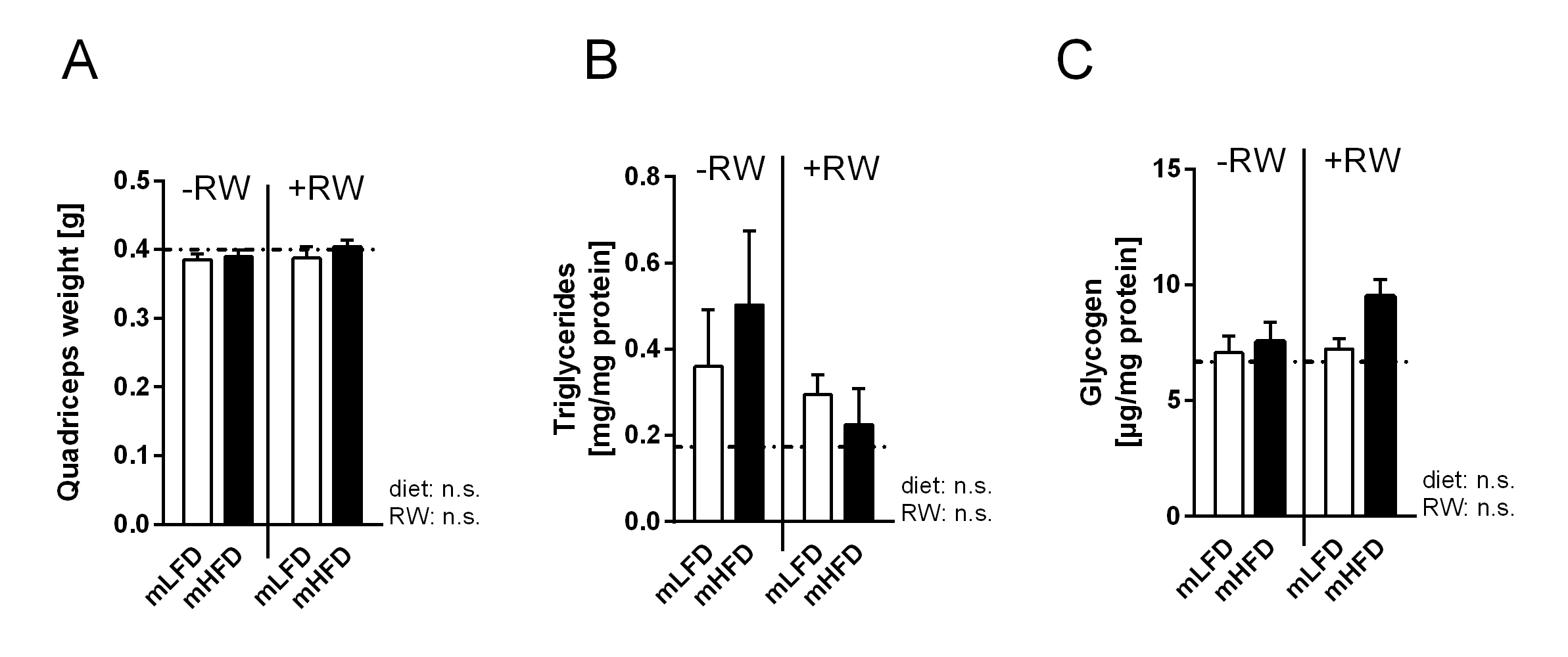

Supplement: S2 Fig — Effects of maternal high-fat consumption on offspring muscle mass (A), triglyceride (B) and glycogen content (C). Maternal low-fat diet (mLFD) or maternal high-fat diet (mHFD) offspring were fed a LFD after weaning throughout an age of 15 wks. Afterwards they received a HFD for 10 wks. Half of each group got access to a running wheel (RW) for voluntary training. The dotted line represents the Con group (mLFD-RW LFD). Data are mean with +SE; (A) n = 10–15; (B/C) n = 4–5. Data were analyzed by two-way ANOVA (Bonferroni post hoc test). (TIF) [file pone.0173076.s004.tif]
